# Comparison of patterns of use, beliefs, and attitudes related to waterpipe between beginning and established smokers

**DOI:** 10.1186/1471-2458-5-19

**Published:** 2005-02-25

**Authors:** Taghrid Asfar, Kenneth D Ward, Thomas Eissenberg, Wasim Maziak

**Affiliations:** 1 Syrian Center for Tobacco Studies, Aleppo, Syria; 2 Department of Health & Sport Sciences, and Center for Community Health, University of Memphis, Memphis, USA; 3 Department of Psychology, Virginia Commonwealth University, Richmond, USA; 4 Institute of Epidemiology and Social Medicine, University of Muenster, Muenster, Germany

## Abstract

**Background:**

To compare patterns of use, beliefs, and attitudes related to waterpipe smoking between university students (beginning smokers) and café customers (established smokers) in Aleppo Syria, in order to explore the evolution of this smoking method.

**Methods:**

Two cross-sectional surveys were conducted among representative samples of university students (total 587, 48.4% men, mean age 22 years), and waterpipe users among cafe' customers (total 268, 60% men, mean age 30 years) in Aleppo, Syria. We used interviewer-administered questionnaire inquiring about pattern of waterpipe smoking (initiation, frequency), situational characteristics of use (partner, place, sharing), beliefs related to waterpipe smoking (harmful/addictive properties of waterpipe), attitudes related to waterpipe smoking (confidence in quitting, will to quit, motivation for quitting, past year quit attempt), and cigarette smoking.

**Results:**

Daily and regular patterns of smoking become more prevalent with increased duration of smoking, but intermittent smoking remains the predominant pattern of waterpipe use. Women seem to be drawn later to the habit, which seem to escape the usual taboo against women's cigarette smoking. Patterns and context of waterpipe use tend to change with progress of the practice affecting frequency, setting, and sharing of waterpipe. Unlike beginners, established waterpipe smokers seem more smoking-method oriented, more hooked on the habit, less willing to quit, and less likely to foresee challenges to quitting.

**Conclusion:**

Use patterns and attitudes related to waterpipe smoking evolve to accommodate the change in dependence and life circumstances of the smoker. Most of use features, beliefs, attitudes, as well as time-course seem unique to this smoking method requiring novel approach to intervention.

## Background

Waterpipe, is a generic name for tobacco use methods that share a common feature; passage of smoke through the water before inhalation. It is known under different names and shapes in different cultures and countries (e.g. shisha, narghile, hookah, hubble bubble) [1]. Although waterpipe smoking is centuries old, its use was declining until recent years when it witnessed a boom in popularity among Arab communities around the world [1,2]. Recent estimates show that this smoking habit has reached a quarter of some groups in the EMR, affecting youths and women among others [3-6].

Studies on the health effects of this smoking method are scanty, and often suffer from poor control of other confounding factors (e.g. cigarette smoking). We are just beginning to learn in a more methodological way the full spectrum of harmful effects of this smoking method and about shared and distinguishing features from cigarette smoking. For example, in regards to CO, one of the main cardiovascular risks of cigarette smoking, waterpipes are at least as efficient as cigarettes in delivering this toxic gas to smokers' lung [1]. On the other hand, tobacco smoke generated by this method seems to contain larger amounts of heavy metals such as arsenic, cobalt, chromium, and lead [7]. In addition, this smoking method is efficient in delivering nicotine to smokers, who tend to show signs of tobacco use dependence [8,9].

Because of the increasing trend of this smoking method, together with its health damaging and addictive potentials the need to develop ways to intervene with smokers and prevent initiation seems timely. Intervention efforts to deal with this emerging public health problem however, are hindered by the inadequacy of data characterizing various aspects related to patterns of waterpipe use and dependence [10].

We recently conducted a survey looking at prevalence, beliefs, and attitudes related to this smoking method among university students in Aleppo, Syria. Waterpipe was practiced by a quarter of male and 5% of female students and was characterized by intermittent and social use [3]. Also, smokers among university students seem to be at the initial stages of their waterpipe practice [3]. Informed by these results, a second survey was carried out among older, more established, smokers in cafés in Aleppo. In this report we compare the two groups in terms of pattern of use, beliefs, and attitudes related to waterpipe smoking in order to gain insights on the natural history of this smoking method and inform intervention development.

## Methods

The methods of these surveys conducted in 2003 are described in details elsewhere [3,9]. Briefly, these surveys were conducted among representative samples of university students (total 587, 48.4% men, mean age 22 years, age range 18–30 years) of whom 86 (15%) were waterpipe smokers, and waterpipe users in coffee shops in Aleppo (total 268, 60% men, mean age 30 years, age range 18–68 years). The students survey was carried out at Aleppo University's dormitories (total 19), where four women's and four men's dormitories were selected randomly, and within each dormitory, a sampling frame was used to recruit about 75 participants. In the cafe' survey, 17 cafes were selected out of total 112 in Aleppo, and within each cafe' participants were recruited by random selection of pre-numbered waterpipes. Unlike the café survey, the university survey included both waterpipe smokers and non-smokers, and thus only waterpipe smokers (15%) among students were included in the current analysis (total 86, 82.5% men, mean age 22 years) (Table 1).

**Table 1:** Basic indicators of the café survey population in comparison to University students

	University students (n= 86) **n (%)**	Café customers (n = 268) **n (%)**
Gender		
Male	71 (82.6)	161 (60,1)
Female	15 (17.4)	107 (39.9)

Residence		
City	43 (50.0)	264 (98,5)
Country	43 (50.0)	4 (1.5)

Religion		
Muslim	81 (94.2)	201 (75.3)
Christian	5 (5.8)	66 (24.7)

Marital status		
Married	1 (1.2)	128 (47.8)
Single, divorced, or widowed	85 (98.8)	140 (52.2)

Frequency of narghile use		
Daily	5 (5.8)	64 (24.0)
Less than daily	81 (94.2)	203 (75.7)

	**Mean ± SD**	**Mean ± SD**

Age	22.3 ± 2.3	30.1 ± 10.2

Economic status (DI)	2.0 ± 0.7	1.2 ± 0.6

Number of years of education	14.7 ± 1.3	12.5 ± 3.6

The questionnaires used in these surveys were developed from standard instruments modified to suit waterpipe smoking [11-13]. The protocol and informed consent document were approved by the Institutional Review Boards at the Syrian Society Against Cancer and The University of Memphis.

Generally, the two groups were compared on the following dimensions; 1- socio-demographics including age, gender, number of years of formal education, economic status assessed by the density index (DI = number of household members/number of rooms in the house), 2- smoking characteristics including frequency of use (daily, occasional), age of initiation of use, age of initiation of daily use, cigarette smoking status (past month cigarette smoking, ex-daily smoking for current non-smokers), 3- situational characteristics of waterpipe use including waterpipe smoking initiation (alone, with friends, with family), current waterpipe smoking (alone, with friends, with family), usual place of waterpipe smoking (open end question categorized later into home/dorms, cafes, other or no particular place), waterpipe sharing with others, usual sharing companion (as an open end question categorized later into friend, family), whether use is periodic/seasonal, the season of increased use (as an open end question categorized later into holidays, summer/spring, stress/exams, and other), 4- quit attitude (waterpipe, cigarette) including belief of own ability to quit, will to quit, past year quit attempt, quit motivation (open end question categorized later into health, cost, and other), perceived major challenge for quitting (open end question categorized into friends, addiction and habit, boredom and free time, no challenge, other or non specific answer), 5- perceived main health hazard of waterpipe smoking (open end question categorized into cardiovascular effects, respiratory effects, cancer, other bodily effects, none or don't know), harmful and addictive effects of cigarettes compared to waterpipe (categorized into cigarettes are more harmful, both methods are equally harmful, and waterpipe is more harmful, and the same categories were used for the comparison of addictiveness), and finally 6- family's attitude towards waterpipe and/or cigarette smoking categorized into (friendly, normal, not friendly), and smokers' perception of the extent they are hooked on waterpipe (categorized into not at all, somewhat, and very hooked) (tables 1, 2, 3). Participants were asked as well on the average time of each waterpipe smoking bout, and daily waterpipe smokers were asked about their average monthly expenditure on waterpipe smoking.

**Table 2:** Waterpipe smoking patterns among café customers (established) compared to those of university students (beginners)

	Beginners (students, n = 86) **n (%)**	Established (customers, n = 268) **n (%)**
Frequency of waterpipe smoking		
Daily	5 (5.8)	64 (24.0%)*
Occasionally	81 (94.2)	203 (76.0)*

First experience with waterpipe smoking		
Alone	2 (2.3)	25 (9.3)*
With friend	69 (80.2)	165 (61.6)*
With family	15 (17.4)	75 (28.0)

Current use of waterpipe		
Alone	1 (1.2)	31 (11.6)*
With friends	69 (80.2)	175 (65.3)*
With family	16 (18.6)	62 (23.2)*

Place of usual waterpipe smoking		
Home	48 (55.8)	51 (19.2)*
Café/restaurant	24 (27.9)	207 (77.8)*
Other or no particular place	14 (16.3)	8 (3.0)*

Share the same waterpipe	83 (96.5)	117 (43.8)*

Individual most commonly waterpipe is shared with		
Friend	78 (94.0)	49 (41.9)*
Family	5 (6.0)	66 (56.4)*

Current cigarette smoking	41 (47.7)	71 (26.5)*

Ex-cigarette smoking	8 (17.8)	24 (12.2)

Seasonal increase in waterpipe smoking frequency	60 (69.8)	131 (48.9)*

Period of increased waterpipe smoking frequency		
Holiday	15 (25.0)	25 (19.1)
Summer	31 (51.7)	91 (69.5)*
Stress/exams	12 (20.0)	5 (3.8)*
Other or no specific answer	2 (3.3)	10 (7.7)

	**Mean ± SD**	**Mean ± SD**

Age of initiation of waterpipe smoking (years)	19.6 ± 2.6	24.3 ± 8.3*

Age of initiation of daily waterpipe smoking (years)	21.8 ± 3.6	24.7 ± 8.8

Duration of waterpipe smoking (years)	2.7 ± 1.9	5.9 ± 6.2*

Monthly cost of waterpipe smoking	680 ± 507	2366 ± 1918*

Age of initiation of daily cigarette smoking (years)	18.8 ± 2.4	19.1 ± 4.1

* *p < 0.05 *using the Chi^2 ^test for dichotomous variables, and Student's *t *test or the Mann-Whitney test as appropriate for continuous variables (comparison is always between beginners and established smokers).

**Table 3:** Attitudes and beliefs related to waterpipe quitting among café customers and university students

	Beginners (students, n = 86) **n (%)**	Established (customers, n = 268) **n (%)**
Belief can quit waterpipe any time	77 (89.5)	231 (86.5)

Will to quit waterpipe	35 (40.7)	76 (28.4)*

Last year quit attempt (waterpipe)	23 (65.7)	45 (59.2)

Belief can quit cigarettes anytime	19 (46.3)	36 (50.7)

Will to quit cigarettes	29 (70.7)	45 (63.4)

Last year quit attempt (cigarettes)	26 (89.7)	32 (71.1)

Main motivation for quitting waterpipe**		
Health	33 (91.6)	79 (103.8)
Cost	3 (8.7)	3 (6.5)
Other	3 (8.6)	4 (5.3)

Main challenge for quitting waterpipe		
Friends	10 (28.6)	6 (7.9)*
Addiction and habit	6 (17.1)	5 (6.6)
Boredom and free time	3 (8.6)	7 (9.2)
No challenge	13 (37.1)	47 (61.8)*
Other or no specific	3 (8.6)	11 (14.5)

Main health hazard of waterpipe		
Cardiovascular effects	5 (5.8)	25 (9.4)
Respiratory effects	41 (47.7)	98 (36.7)*
Cancer	25 (29.1)	96 (36.0)
Other bodily effects	11 (12.7)	13 (4.8)*
None, don't know, none specific	4 (4.7)	35 (13.0)*

Belief about the addictive effects of waterpipe compared to cigarettes		
Cigarettes are more addictive	77 (89.5)	221 (82.8)
Equally addictive	7 (8.1)	27 (10.1)
Waterpipe is more addictive	2 (2.3)	19 (7.1)

Belief about harmful effects of waterpipe compared to cigarettes		
Cigarettes are more harmful	32 (37.6)	124 (46.4)
Equally harmful	11 (12.9)	49 (18.4)
Waterpipe is more harmful	42 (49.4)	94 (35.2)*

* p < 0.05 using the Chi^2 ^test ** Multiple responses were allowed (percentages add to more than 100)

### Statistical analysis

Frequency analyses of main study indices were tabulated and differences between the two groups were assessed using the Chi^2 ^test for dichotomous variables. Measures of central tendency were calculated for continuous variables, expressed as mean ± standard deviation (SD), and were compared between the two groups using Student's *t *test or the Mann-Whitney test as appropriate. Spearman correlation coefficient was calculated for the relation between duration of waterpipe smoking and frequency of use (categorized as daily, weekly, monthly) in the café study. SPSS statistical software (release 11) was used for the analysis with *p *value <0.05 considered significant.

## Results

Overall, the two samples combined show that daily waterpipe smoking was seen among 19.5% of participants (23.3% of smoking men and 12.3% of smoking women), while occasional smoking was seen among 80.2% of participants (76.3% of smoking men and 87.7% of smoking women). More than half of waterpipe users initiated use and currently use waterpipe with a friend (Table 2). About a quarter of waterpipe users (26.5%) smoke cigarettes as well. Duration of use and frequency of use (daily, weekly, monthly) were correlated (Spearman correlation coefficient 0.14, *p = 0.02*). Mean duration of waterpipe smoking session among café customers was 71.1 ± 35.8 minutes.

Comparison of patterns of initiation and current use of waterpipe between café customers and university students is detailed in Table 2. Mainly, café customers initiate waterpipe use and daily use later and have longer duration of waterpipe smoking compared to students. University students were more likely to start, currently smoke, and share the same waterpipe with their friends compared to their café counterparts (Table 2). Also, a marked difference between café customers and students is that cigarette smoking was more common among students waterpipe smokers than café ones (*p < 0.01*), still ex-cigarette smoking did not differ between the two groups. Students as well were more likely to have a seasonal pattern of waterpipe use, which was more associated with exams and stress compared to café customers (Table 2).

Quit attitude of café customers shows that the majority (86.5%) believe that they can quit waterpipe any time, but only a minority (28.4%) are interested in quitting, mainly out of health motivation (Table 3). In comparison, interest in quitting was higher among students and friends were more likely to be cited as the main challenge to quitting (Table 3). This was different from cigarette smokers, where only about a half of those (in both groups) believed they can quit anytime and the majority were willing to quit. Waterpipe users among café customers equally acknowledge respiratory disease and cancer as associated with waterpipe smoking.

**Figure 1:**
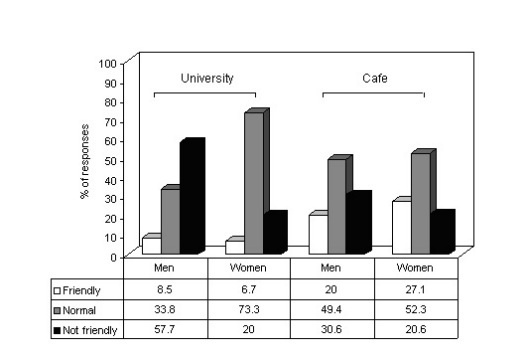
Family attitude towards participants (café customers, university students) waterpipe smoking showing more tolerant attitude for women's waterpipe smoking compared to men's. This unique observation marks the first incident in this region where a smoking method by women is more tolerated than by men.

**Figure 2:**
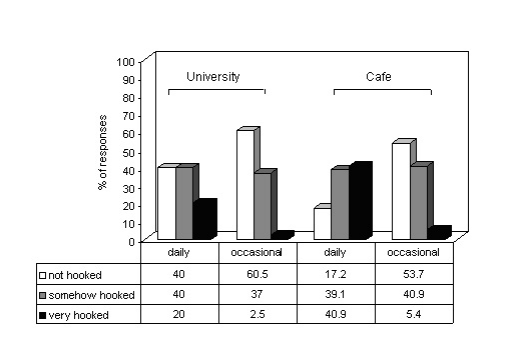
Self-perceived dependence among waterpipe smokers according to their frequency of waterpipe use (café customers compared to students). It shows that dependence is related to frequency of use, and that at comparable level of use, established smokers among café customers are more likely to perceive themselves as being hooked on the waterpipe compared to students.

Attitude of families towards participants' waterpipe smoking for both café customers and students are depicted in figure 1, showing more tolerance for women waterpipe smoking than for men's in general. Figure 2 illustrates participants (both groups) self perception of being hooked on waterpipe according to their frequency of smoking, were these dimensions were strongly associated among café customers but not students (*p < 0.001*, *p = 0.1*, respectively).

Finally, both mean education years and DI differed significantly between café customers (mean education years12.5 ± 3.6; mean DI 1.2 ± 0.6) and students (mean education years 14.7 ± 1.4, mean DI 2.0 ± 0.7) (*p < 0.01 *for both).

## Discussion

Obviously café customers are different from university students in many attributes that can reflect on waterpipe smoking and attitudes towards it. The longer duration of waterpipe smoking of café customers compared to students suggests that these two groups have been sampled at different stages of their waterpipe smoking practice. As such the two studied groups can provide an opportunity to look at features relevant to initiation and maintenance of waterpipe smoking. Such information can advance our understanding of this unique and increasingly popular smoking method and aid the design of intervention strategies. Generally, it looks that smoking patterns and quit attitude are mostly shaped by the stage of smoking, while beliefs and knowledge related to waterpipe tend to follow a more subtle roots likely reflecting cultural and socio-economic attributes.

The first important characteristic that differentiates waterpipe use patterns in the two studied groups is that daily use was much higher among café customers practiced by about a quarter of waterpipe smokers. This is likely to reflect the time-course of waterpipe smoking, with regular use becomes more noticed as duration of smoking increases. In fact duration of waterpipe smoking was correlated to frequency of smoking (daily, weekly, and monthly) among café customers. Still, unlike cigarette smoking, which is practiced mainly on a daily basis in the two studied populations and in the Syrian society in general [13], intermittent use seems to be the predominant pattern of waterpipe smoking. This observation is supported by data from other countries in the EMR highlighting the unique social dimension of waterpipe use [4,14]. However, with average smoking bout of more than one hour and the potentially high levels of different toxicants in waterpipe smoke (arsenic, cobalt, chromium, lead) [7], even intermittent use can be associated with significant health hazards to smokers.

Age of initiation of waterpipe differed significantly between men and women for both studied groups (analysis not shown). This may explain why the proportion of women among café customers is double that of students, although one should bear in mind the limitation of the samples for such a comparison. We have to be mindful as well that unlike cigarette smoking we are still at the expansion stage of the waterpipe epidemic, where people of different age groups are joining the new hype at relatively the same time period. An indication of this fact is that while age of initiation of waterpipe differed between the two studied groups, age of initiation of daily cigarette smoking was somehow identical. The tolerant attitude towards waterpipe smoking by women can aid its spread among them (Figure 1) [3,15]. Being perceived as closer to the local traditions, waterpipe smoking may escape the societal taboos of cigarette smoking by women in the EMR [15,16].

The social dimension, which is a salient feature of waterpipe smoking, seems also to differ between the two studied groups. So while students mainly initiate and currently practice the habit with their friends, more smokers among café customers are initiating or currently practicing the habit with family members or alone, despite being sampled in a social setting. It appears that the social context of waterpipe smoking changes with the change of both personal and smoking related characteristics (marriage, increased dependence). Our previous analysis of factors related to frequency of waterpipe use, as a marker of dependence, has lead us to suggest that as waterpipe smokers become more dependant the social pattern of smoking is gradually replaced with more individual one [9]. Sharing the same waterpipe and seasonal increase of waterpipe smoking, another important features of waterpipe smoking among students, were less noticed among café customers. It is likely that as waterpipe smokers move from the phase of experimentation to regular use they become more dependant, as such the habit is increasingly practiced in a way to satisfy dependence rather than as a pure social utility. Noticeably, at comparable level of use (daily) café customers perceive themselves to be more hooked on waterpipe than students (Figure 2).

Waterpipe quit attitude and perceived challenges to quitting differed between café customers and university students, with café customers demonstrating lower interest in quitting and lower perception of challenges to quitting (Table 3). This can be related to the fact that while students waterpipe smokers come mainly from cigarette smokers (with higher cigarette smoking levels compared to their peers, 3), waterpipe smokers among café customers seem to be more smoking method-oriented (with lower cigarette smoking levels than that of adults in Syria, 13). The fact that ex-cigarette smoking did not differ between the two groups supports this argument. In addition, café customers were in disagreement with students about which smoking method is more harmful, favoring the waterpipe (less harmful). Finally, waterpipe smokers in cafés seem to be more hooked on this smoking method compared to students, and dependence among café customers was inversely related to their willingness to quit [17]. Thus, the difference of quit attitude between the two groups is likely to different stage and orientation along waterpipe smoking practice. The predominance of intermittent use among both groups on the other hand, may have created false perception of easiness of quitting waterpipe. This is likely to be a false perception as about two thirds of those willing to quit waterpipe in both groups made an unsuccessful quit attempt last year. In fact unlike the long held belief linking dependence and difficulty in quitting to daily/frequent use, new studies among youth cigarette smoking suggests that dependence and difficulty in quitting can develop at low levels of consumption [18,19].

As mentioned above a limitation of this study is that the café sample cannot be considered representative of adult smokers in the community. Also, the study consists of two cross sectional surveys in two populations presumably at two different stages of their waterpipe practice. Still, the two groups are not totally distinct from each other as they overlap in terms of age and smoking characteristics. However, as noted from the discussion we are mindful of these limitations throughout analyses and interpretation of the study's results.

This study shows the predominance of intermittent use of this smoking method as well as its increasing popularity among women who are drawn to the habit relatively later than men. The social context of waterpipe smoking, which is a defining feature of this smoking method, tends to change to accommodate smoker's life and dependence progress. Our data suggest that at early stages of waterpipe use, users tend to come from cigarette smokers or people with liberal attitude towards smoking in general, but at more advanced stages of use smokers tend to be more smoking method-oriented and less keen on quitting. There is a general belief of easiness to quit waterpipe compared to cigarette, but this is likely to be an under-estimation as it is not translated into high success rate of quit attempts. Intervention or prevention strategies to curb this emerging epidemic should take into consideration the unique use features of this smoking method as well as smokers' perceptions and attitudes towards it.

## Competing interests

The author(s) declare that they have no competing interests.

## Authors' contributions

TA designed the study and wrote the first draft. KDW and TE participated in the study design and co-authored the manuscript. WM participated in the study design and wrote the final draft.

## Pre-publication history

The pre-publication history for this paper can be accessed here:


